# Higher gene expression stability during aging in long-lived giant mole-rats than in short-lived rats

**DOI:** 10.18632/aging.101683

**Published:** 2018-12-16

**Authors:** Arne Sahm, Martin Bens, Yoshiyuki Henning, Christiane Vole, Marco Groth, Matthias Schwab, Steve Hoffmann, Matthias Platzer, Karol Szafranski, Philip Dammann

**Affiliations:** 1 Leibniz Institute on Aging – Fritz Lipmann Institute, Jena, Germany; 2 Department of General Zoology, Faculty of Biology, University of Duisburg-Essen, Essen, Germany; 3 Institute of Physiology, University of Duisburg-Essen, 45147 Essen, Germany; 4 Department of Neurology, Jena University Hospital-Friedrich Schiller University, Jena, Germany; 5 University Hospital, Central Animal Laboratory, University of Duisburg-Essen, Essen, Germany; * Shared senior authorship

**Keywords:** Fukomys, Bathyergidae, differential gene expression, longevity, collagen

## Abstract

Many aging-associated physiological changes are known to occur in short- and long-lived species with different trajectories. Emerging evidence suggests that numerous life history trait differences between species are based on interspecies variations in gene expression. Little information is available, however, about differences in transcriptome changes during aging between mammals with diverging lifespans. For this reason, we studied the transcriptomes of five tissue types and two age cohorts of two similarly sized rodent species with very different lifespans: laboratory rats (*Rattus norvegicus*) and giant mole-rats (*Fukomys mechowii*), with maximum lifespans of 3.8 and more than 20 years, respectively. Our findings show that giant mole-rats exhibit higher gene expression stability during aging than rats. Although well-known aging signatures were detected in all tissue types of rats, they were found in only one tissue type of giant mole-rats. Furthermore, many differentially expressed genes that were found in both species were regulated in opposite directions during aging. This suggests that expression changes which cause aging in short-lived species are counteracted in long-lived species. Taken together, we conclude that expression stability in giant mole rats (and potentially in African mole-rats in general) may be one key factor for their long and healthy life.

## Introduction

Compared to short-lived mammals, long-lived mammals have repeatedly been shown to exhibit fewer age-associated changes in numerous physiological parameters related to the functional decline during aging [1–4]. Recent RNA-seq studies have suggested that much of the remarkable lifespan diversity among mammals is based on interspecies differences in gene expression [5,6]. However, those studies focused on identifying particular genes and pathways that are differentially expressed between species with divergent longevities. Whether short- and long-lived species differ at the transcript level with respect to their amount of differentially expressed genes (DEGs) during aging (hereinafter referred to as “gene expression stability”) has, to the best of our knowledge, not been explored yet.

Here, we examined age associated transcriptome changes in two similarly sized rodent species with different longevities: the laboratory rat (*Rattus norvegicus*), which has a maximum lifespan of 3.8 years [7], and the giant mole-rat (*Fukomys mechowii*), which has a maximum lifespan of more than 20 years ([8] and own unpublished data). In giant mole-rats, longevity is significantly correlated with the reproductive status. Breeding animals outlive non-breeders by far [8]. In the current study, we examined only non-breeding males. Male non-breeding giant mole-rats have a maximum lifespan of approximately 10 years and an average lifespan of approximately 6 years, still clearly exceeding the life expectancy of the laboratory rat [8]. For both species, we performed RNA-seq on tissue samples from five organs (blood, heart, kidney, liver, and skin; hereinafter called simply tissues) of young and elderly adults. The tissues were collected from young and elderly cohorts of laboratory rats (0.5 and 2.0 years) and giant mole-rats (young: approximately 1.5 years at average; elderly: approximately 6.8 years at average; see Tables S1-S3 for details). For both species, the first time points were chosen to sample young, sexually mature adults. The second time points correspond to an age-associated survival rate of less than 40% in rats and giant mole-rats (Tables S1-S3) [8,9]. For each species, we determined DEGs between the two respective time points and searched for enriched functional categories.

## RESULTS

**Figure 1:**
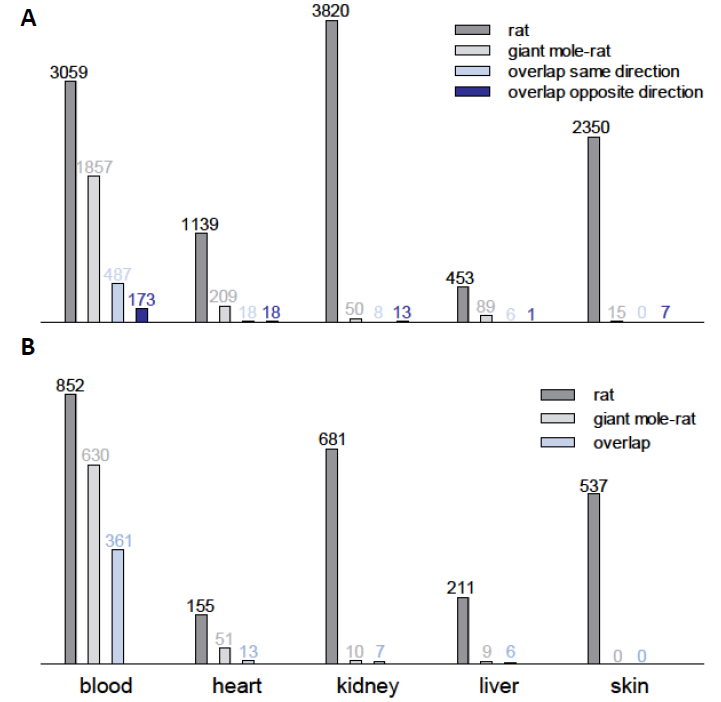
**Comparative transcriptomics in giant mole-rats and laboratory rats of elderly vs. young individuals.** (**A**) Counts of differentially expressed genes (DEGs) during aging in five tissue types from laboratory rats (*Rattus norvegicus*) and giant mole-rat (*Fukomys mechowii*). Only orthologous genes in both transcript catalogs were counted (n=14,062). (**B**) Numbers of biological processes (Gene Ontology) enriched for DEGs during aging in five tissue types from laboratory rats (*R. norvegicus*) and giant mole-rats (*F. mechowii*).

The giant mole-rat transcriptomes changed much less during aging (Tables S4-S13). In four of five tissue types, the number of orthologous DEGs in the giant mole-rats was only a fraction of the respective number in the laboratory rats (0.6%-19.0%; Fig. 1a). The number of DEGs was similar only in the blood of both species but still was 40% lower in blood from the giant mole-rats than in blood from the laboratory rats. Across tissues, the giant mole-rat transcriptomes contained significantly fewer DEGs during aging than did the laboratory rats (*P* = 0.016, Wilcoxon signed-rank test).

To ensure that the low number of identified DEGs in the giant mole-rat was not caused by a low statistical power compared to the rat, we examined the statistical power per species and tissue using the RNA-seq data dispersion [10]. We estimated a statistical power of 83-95% and 80-90% in giant mole-rat and rat, respectively, depending on the tissue (Table S14). Thus, this finding corroborates our evidence that the lower number of detected DEGs in the giant mole-rat might indeed reflect a greater expression stability during aging. Furthermore, we ensured that there was no relevant difference in the measured gene expression levels between the examined species (Fig. S10, all DEGs). Those genes, however, that were found to be differentially expressed in both species (Fig. S10, overlapping DEGs), tended to be lower expressed in the rat and higher expressed in the giant mole-rat, compared to median across all DEGs.

Upon Gene Ontology [11] analysis of the differentially expressed genes, we found typical molecular aging signatures across all examined tissues in the rat (Fig. 1b). For instance, altered expression levels of immune response genes (Gene Ontology [GO]:0006955; Tables S15-S24) and inflammatory response genes (GO:0006954) are known to be hallmarks of aging [12]. These, as well as many related processes, such as response to cytokine (GO:0034097) and leukocyte aggregation (GO:0070486), were consistently enriched for DEGs in all examined laboratory rat tissues. In the giant mole-rat, on the other hand, we found these signatures only in blood.

**Figure 2:**
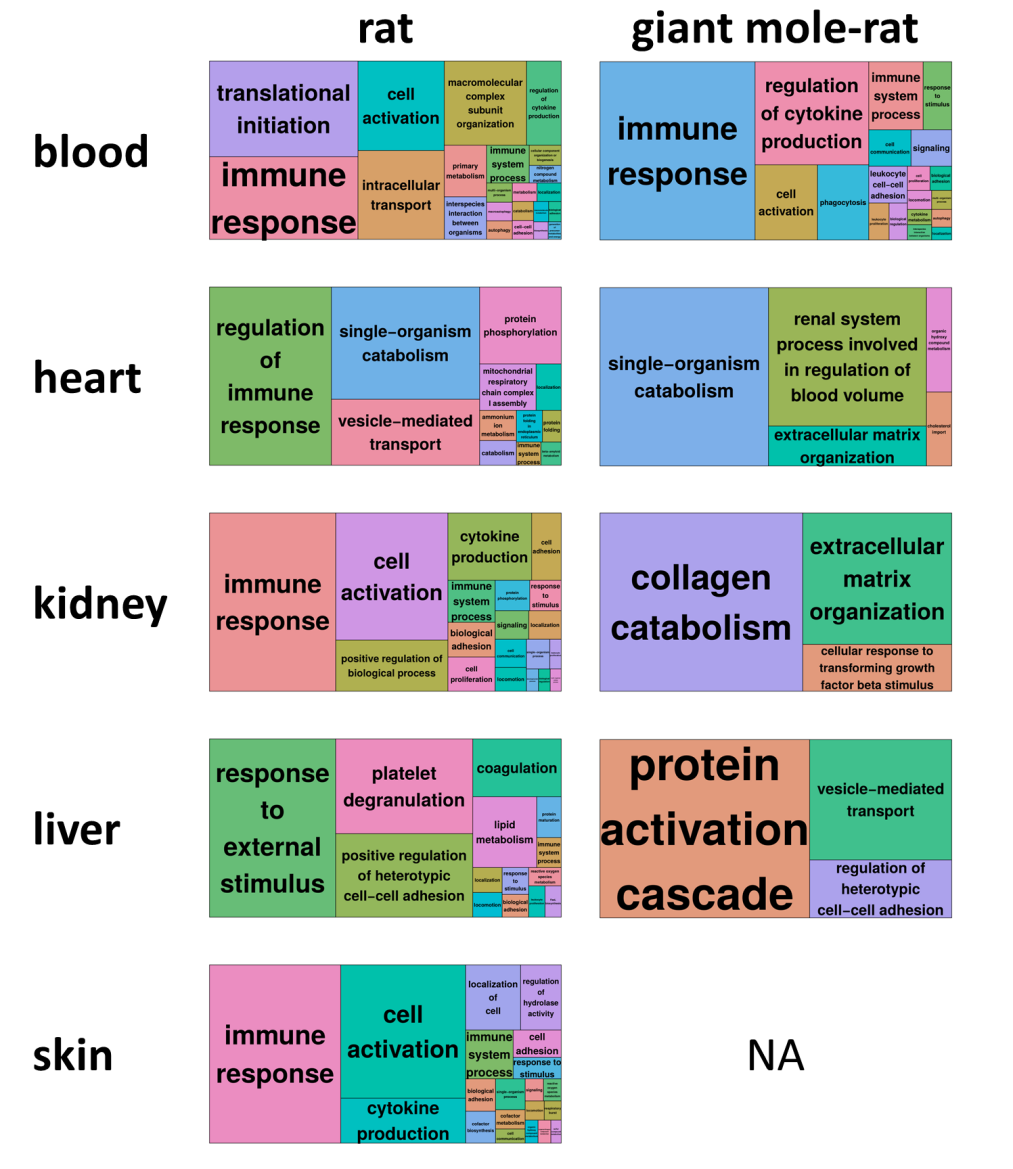
**REVIGO treemap summary of gene ontology processes that are significantly enriched (false discovery rate [FDR] < 0.05) for differentially expressed genes during aging.** For each species and tissue, the superclusters, *i.e*., the highest summarization level of gene ontology processes, as identified by REVIGO [13] are shown. Each rectangle painted with a unique color represents a supercluster. The colors only serve to distinguish superclusters. The size of the rectangles represents their p-value, *i.e*., largest rectangles represent the most significant superclusters. For giant mole-rat skin, no treemap could be generated since no gene ontology process was significantly enriched (Fig. 1b). Corresponding REVIGO treemap summarizations are provided as high-resolution Figures S1-S9, showing also the clusters within the superclusters.

The clustering of DEG enriched biological processes with REVIGO [13] revealed that immune-related functions, immune process or regulation of immune process, determine the largest superclusters in four of five rat tissues (Fig. 2, Fig. S1-S9). Additional aging-relevant clusters found across rat tissues were apoptotic process (GO:0006915; all tissues except heart), coagulation (GO:0050817; all tissues) and oxidation-reduction process (GO:0055114; all tissues except liver). Except in blood, the giant mole-rat did not exhibit the same (or similar) DEG enriched biological processes. These findings indicate typical aging-dependent gene expression alterations are slowed down in several vital tissues of giant mole-rats.

On the single gene level, there was a modest but still statistically significant (*P* < 0.05; Fisher’s exact test) overlap between the DEGs of laboratory rats and those of giant mole-rats in blood, heart, and skin tissues (Fig. 1a; Tables S25-S29). Common DEGs in the blood of laboratory rat and giant mole-rat were often regulated in the same direction (up or down) during aging in both species (*P* = 3.3*10^-31^; Fisher’s exact test based on regulation of all genes). This finding matches the shared aging signatures (DEG function analysis, see above) in this tissue. Interestingly, in skin samples we found a contrasting overrepresentation of DEGs, which are regulated in opposite directions (*P* = 0.005). This finding points to the intriguing possibility that, in some tissues, expression changes that cause aging in the laboratory rat are counteracted by opposite changes during aging in the giant mole-rat.

In kidney tissues, most shared DEGs were regulated in opposite directions between species during aging (Fig. 1a). As an example, collagen metabolic process (GO:0032963) is one of the seven processes that are enriched in the kidneys of both laboratory rats and giant mole-rats. Although the enrichment in the laboratory rat was based on 20 collagen genes that were significantly up-regulated and one that was down-regulated during aging, in the giant mole-rat this enrichment resulted from four collagens and two genes that code for potent collagenases (*CTSK* and *CTSS*), all of which were down-regulated during aging. Of these six collagenases and collagens, five overlapped with those, which were significantly up-regulated in laboratory rats. Collagen regulation reflects well the molecular aging process because decreasing collagen levels attenuate kidney diseases in rats [14], whereas increased collagen levels in the kidney have been shown to induce the development of cysts in rats with polycystic kidney disease [15]. At the same time, kidney diseases are an important cause of death in rats [16] and perhaps also in (naked) mole-rats [17]. The opposite collagen regulation pattern in the giant mole-rat can be interpreted as an anti-aging program rather than as a signature of the aging process.

## DISCUSSION

The gene expression stability of giant mole-rats during aging that we show here concurs with a general pattern of stability. This has emerged from numerous molecular and physiological comparisons of the extremely long-lived naked mole-rat (*Heterocephalus glaber*, a close relative of giant mole-rats) with shorter-lived species of mice or rats: For example, during aging, naked mole-rats maintain an unchanged membrane lipid composition [3], a fairly stable production of reactive oxygen species [18], and relatively stable levels of oxidative damage to lipids [2], as well as high protein stability and integrity [19]. At the same time, all of these variables, which are known to be among the key factors for lifespan and age-related diseases [20], change significantly in an unfavorable direction during aging in short-lived mice or rats. Naked mole-rats also exhibit minimal decline of physiological functions and maintain activity, fertility, and body composition into old age; they are also remarkably resistant to cancer, and their cancer-associated mortality rates do not increase substantially with age [1]. Given that naked- and giant mole-rats are closely related [21], our own husbandry experience with giant mole-rats leads us to assume that several of the aforementioned properties are shared by both species.

In line with our results, an earlier study showed that gene expression in three types of tissue from naked mole-rats remains nearly unchanged during the first half of their lifespan [22]. However, the statistical power of this analysis was very limited because the study used only one replicate per age. Regarding laboratory rats, our results are in good agreement with the findings of the rat body map initiative [23]. This database shows many DEGs (491 to 14,062) across eleven types of tissue during rat aging; the time points used in this study are similar to ours (21 weeks vs. 2 years). The results of Kim et al. [22] and of the rat body map project cannot be directly compared with each other because those studies used different methods for sequencing and DEG detection. Therefore, in this study both species were examined with the same sequencing procedure and the same bioinformatic analyses. Thus, we confirmed that, the gene expression of a long-lived African mole-rat species - in contrast to those of a short-lived rodent - indeed remains stable during aging from young to a elderly adulthood. Since gene expression is a basic regulatory process of the cell that determines many of the above-mentioned molecular phenotypes and physiological observations, we suggest that gene expression stability during aging is one of the key causal factors for the extraordinary long and healthy lifespan of this African mole-rat species, and potentially of the whole family.

In conclusion, we hypothesize that the higher gene expression stability observed in long-lived giant mole-rats compared to short-lived rats evolved under different evolutionary constraints and contributes to the considerably distinct life history traits of the short- and long-lived species: early onset and fast aging in one species, and delayed or slowed aging from youth to elderly adulthood in the other.

## MATERIALS AND METHODS

### Experimental design

This study compared the transcriptomes of young and elderly animals from two species: Wistar rats (*Rattus norvegicus*) and giant mole-rats (*Fukomys mechowii*). Samples from five tissues (blood, heart, kidney, liver, and skin) were taken from animals in both species and both age cohorts. All examined animals were non-breeding males. Young and elderly laboratory rats were 6 and 24 months of age, respectively, and sampled in April, October and November 2016 (see Table S1 for details). Library preparation and sequencing was performed for all but three rat samples in one batch in December 2016 – the remaining three were sequenced in January 2017 (Table S3). Young mole-rats were 1.3 to 2.0 years old (grand mean across tissues: 1.5 years), whereas elderly mole-rats were 5.5 to 7.7 years old (grand mean across tissues: 6.8 years). Mole-rats were sampled in 5 distinct sampling sessions between February 2014 and December 2016 (Table S1). Sequencing of mole-rat samples was performed in 7 runs across the same time frame (Table S3).

We examined samples from 4 to 8 animals per tissue for each age cohort and species (Tables S1-S3). All animals were healthy at the time when they were sacrificed.

For tissue collection, rats were euthanized with CO_2_. Mole-rats were anaesthetized with 6 mg/kg ketamine combined with 2.5 mg/kg xylazine and then euthanized by surgical decapitation. Immediately after dissection, tissue samples were transferred to tubes containing RNA-protective buffers and stored in -80°C until analysis.

For both species, the first age group consists of young, sexually mature adults. Their age was approximately one-fourth of the second group’s age, which corresponds to a survival fraction of approximately 39% and 24% in rats and giant mole-rats, respectively (Tables S1-S3) [8,9]. In relation to maximum lifespan the median age at the second time point represents 53% in rats (maximum lifespan in male *Rattus norvegicus*: 3.8 years) and 68% (maximum lifespan in *Fukomys mechowii* non-breeders: 10 years) [7,8]. Thus, the chosen time points represent similar biological ages in the examined species with a wider age-range between the compared time points in giant mole-rats. The latter means that the observed smaller age-related changes of the transcriptomes in giant mole-rats compared to rats are conservative findings.

For tissue collection, rats were euthanized with CO_2_. Mole-rats were anaesthetized with 6 mg/kg ketamine combined with 2.5 mg/kg xylazine and then euthanized by surgical decapitation.

Animal housing and tissue collection was compliant with national and state legislation (breeding allowances 32-2-1180-71/328 (mole-rats) and 32-2-11-80-71/345 (rats), both Ordnungsamt Essen, Northrhine-Westfalia, Germany).

### Transcript catalogue sequences

The giant mole-rat transcript catalog was assembled and annotated with human gene symbols on the basis of recently published read data [24] (European Nucleotide Archive [ENA] study PRJEB20584) and the assembly framework FRAMA [25] with default parameters. For laboratory rats, mRNA sequences were obtained from NCBI RefSeq. Ortholog relations between rat and human genes were downloaded from Ensembl Biomart. For both species, only the longest transcript isoform per gene was used, which is the method of choice for selecting a representative variant in large-scale experiments [26]. This resulted in 15,864 reference transcripts (genes) for the giant mole-rats and 23,479 reference transcripts (genes) for the laboratory rats of which 14,062 reference transcripts (genes) were annotated with the same human gene symbol.

### RNA-seq, read mapping and quantification

Tissue samples were collected and stored in RNAlater (Qiagen, Venlo, Netherlands) after isolation. For all tissues except blood, RNA was purified with the RNeasy Mini Kit (Qiagen) according to the manufacturer’s protocol. Blood samples (100 µl) were collected in RNAprotect Animal Blood reagent (Qiagen). The resulting RNA was purified with the RNeasy Protect Animal Blood Kit (Qiagen). Kidney and heart samples were treated with proteinase K before extraction, as recommended by the manufacturer. Poly(A) selection and preparation of the RNA-seq libraries was performed with the TruSeq RNA v2 kit (Illumina, San Diego, USA). RNA-seq was performed by single-end sequencing with 51 base pairs on a HiSeq 2500 sequencing system (Illumina) and with at least 17 million reads per sample, as described in Table S3. The reads were aligned to the respective reference – rat or giant mole-rat (see above) – with the BWA aln algorithm of the Burrows-Wheeler Aligner (BWA) [27], allowing no gaps and a maximum of two mismatches in the alignment. Only those reads that could be uniquely mapped to the respective gene were used for quantification.

Read data for rats and giant mole-rats were deposited as ENA study PRJEB23955 (Table S3). Read counts per gene and sample can be found in Tables S3 and S31.

### Method validation

To ensure the reliability of our RNA-seq results we determined pairwise Pearson correlation coefficients between all rat and all giant mole-rat samples, respectively, based on log-transformed read counts that were normalized for sample size (Tables S32, S33). For each species and tissue, we calculated the means and standard deviations (Table S34). The grand mean of the determined correlation coefficients across tissues was 0.96 and 0.97 for rat and giant mole-rat, respectively, and the mean standard deviation across tissues 0.02 for both species.

Furthermore, we estimated the statistical power of DESeq2 [28] based on the respective dispersion in our complete rat and giant mole-rat data sets, respectively, using the method of Ching et al. [10] and 10 simulation runs per species and tissue (Table S14).

### Differential expression analysis

Differential expression analysis was performed with DeSeq2 [28]. In both species, the elderly animals were compared with their young conspecifics. Genes that showed a comparison p-value less than 0.05 after Benjamini-Hochberg correction for multiple testing were considered as DEGs. Initial numbers of DEGs per tissue and species were as follows: 4033 and 2002 (blood), 1506 and 227 (heart), 5015 and 57 (kidney), 635 and 94 (liver), 3231 and 18 (skin) DEGs were identified in rat and giant mole-rat, respectively (Tables S4-S13).

To acquire comparable numbers of DEGs and to determine the amount of DEGs that were found in both species (overlap), only those genes were taken into account that were present in the transcript catalogs of both species based on human gene symbol annotation (Fig. 1a, n=14,062). The giant mole-rat transcript catalog was annotated against human (see above). Ortholog relations between rat and human were downloaded from Ensembl Biomart.

Biological processes that were enriched for DEGs were determined in both examined species by using the human gene symbol annotation of the DEGs (see above), their human gene ontology annotation (GO; annotation package: org.Hs.eg.db) and Fisher’s exact test. The Benjamini-Hochberg method was used to correct the resulting *p*-values for multiple testing. Additionally, GO categories with a *p*-value of less than 0.05 after corrections for multiple testing were summarized with REVIGO (cutoff, 0.70; measure, SimRel; database, whole Uniprot) [13] (Fig. 2, Fig. S1-S9).

## SUPPLEMENTARY MATERIAL

Supplementary Figures S1-S10

Supplementary Tables S1-S34
