# Supplementary material for: Higher gene expression stability during aging in long-lived giant mole-rats than in short-lived rats
Source: Aging (Albany NY). 2018 Dec 16;10(12):3938–56. doi: 10.18632/aging.101683 (PMC6326690; doi:10.18632/aging.101683)
Supplement: Supplementary Figures S1-S10 [file aging-10-101683-s001.pdf]

Supplementary figures

Figures S1-S9. REVIGO treemaps of gene ontology processes that are significantly enriched (false discovery rate [FDR] < 0.05) one figure for tissue and species; figure for giant mole-rat skin is missing because the number of enriched terms was too small for summarizing).
Figure S10. Expression levels of examined genes.

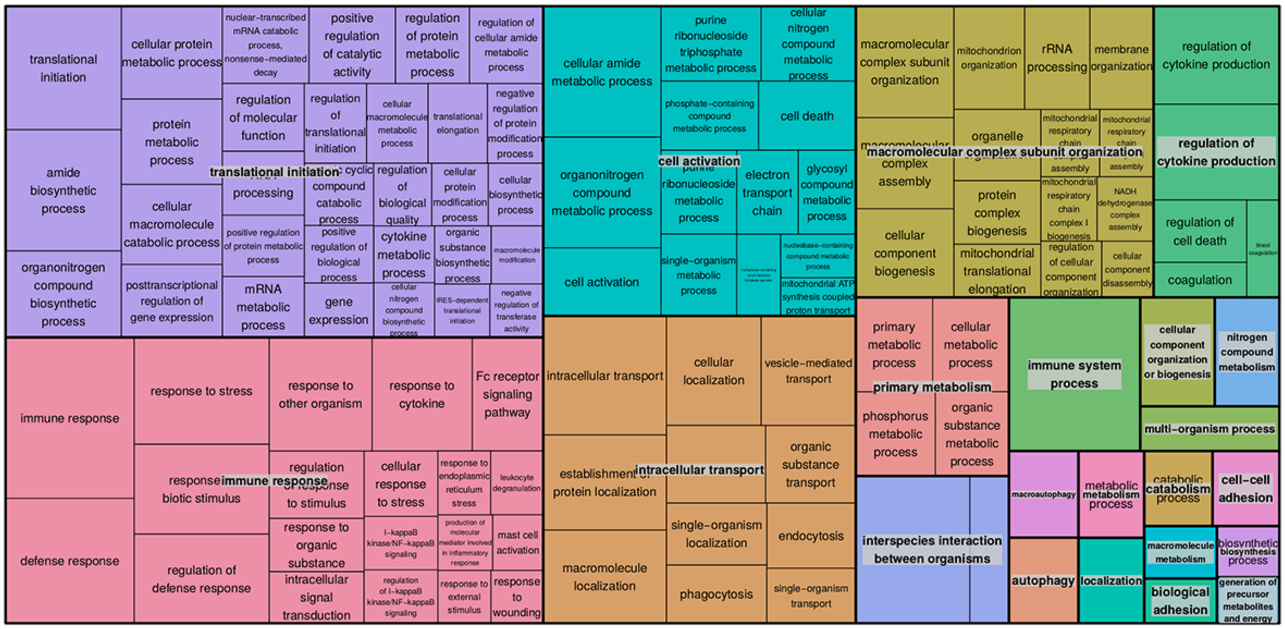

Figure S1. REVIGO treemap of gene ontology processes that are significantly enriched (FDR<0.05) for gene ontology processes in rat blood. Each rectangle is a single cluster representative. The representatives are joined into superclusters of loosely related terms, visualized with different colors. The size of the rectangles reflects the enrichment p-values of the covered GO-terms.

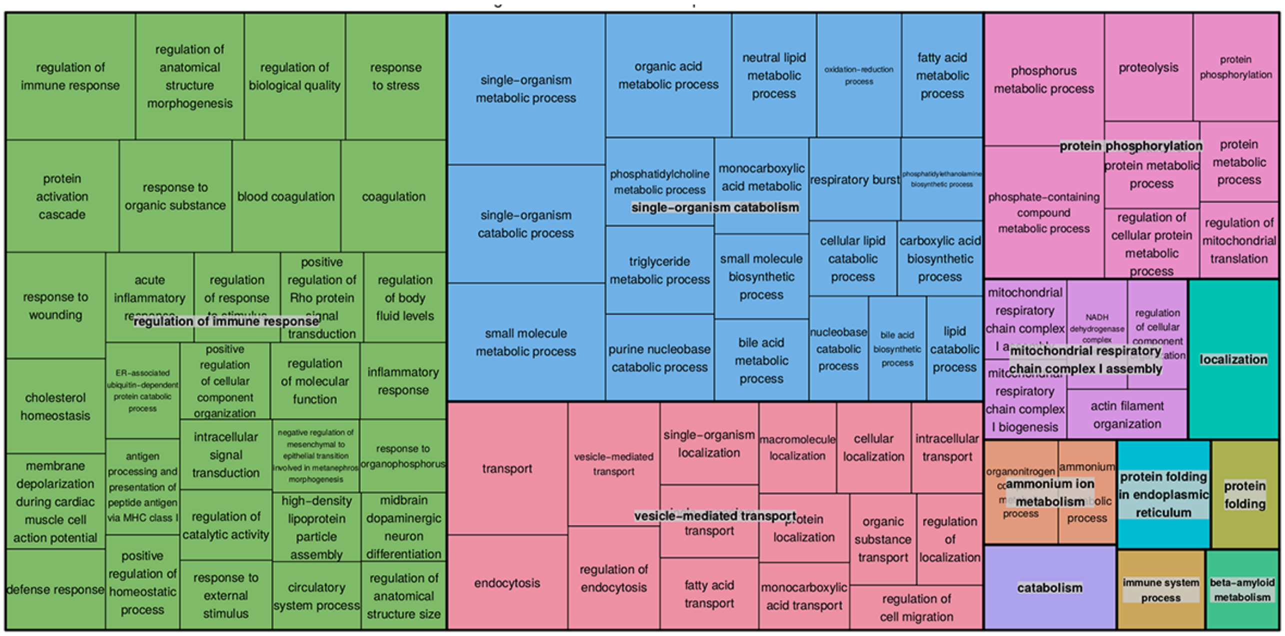

Figure S2. REVIGO treemap of gene ontology processes that are significantly enriched (FDR<0.05) for gene ontology processes in rat heart. Each rectangle is a single cluster representative. The representatives are joined into superclusters of loosely related terms, visualized with different colors. The size of the rectangles reflects the enrichment p-values of the covered GO-terms.

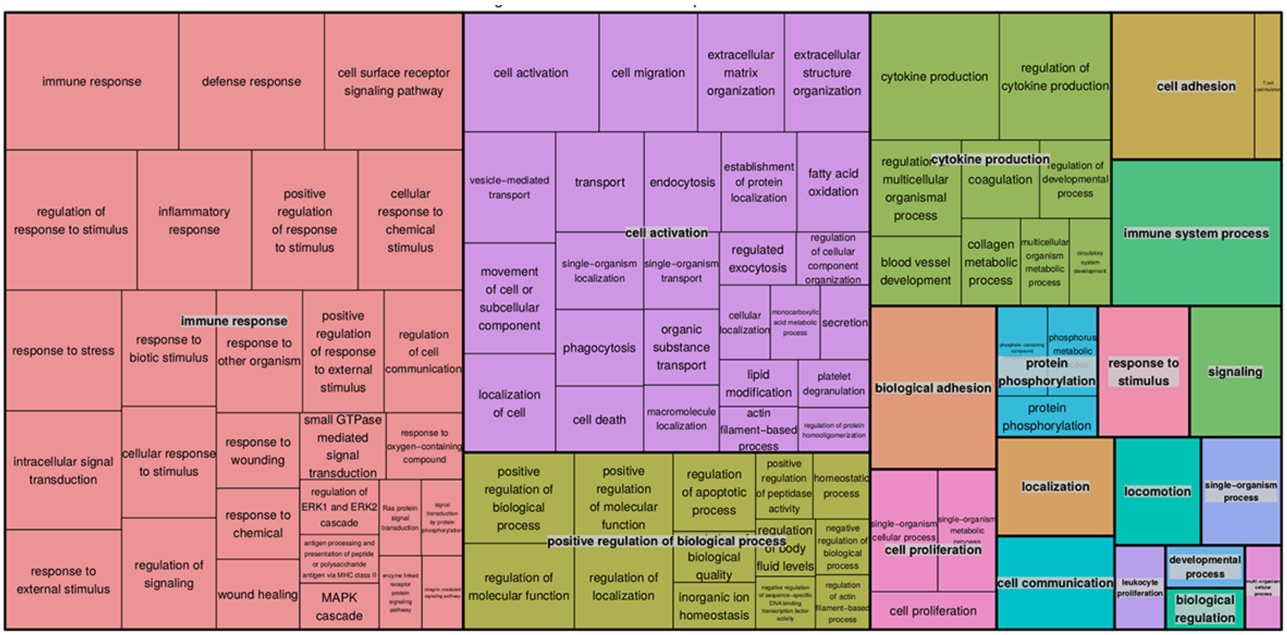

Figure S3. REVIGO treemap of gene ontology processes that are significantly enriched (FDR<0.05) for gene ontology processes in rat kidney. Each rectangle is a single cluster representative. The representatives are joined into superclusters of loosely related terms, visualized with different colors. The size of the rectangles reflects the enrichment p-values of the covered GO-terms.

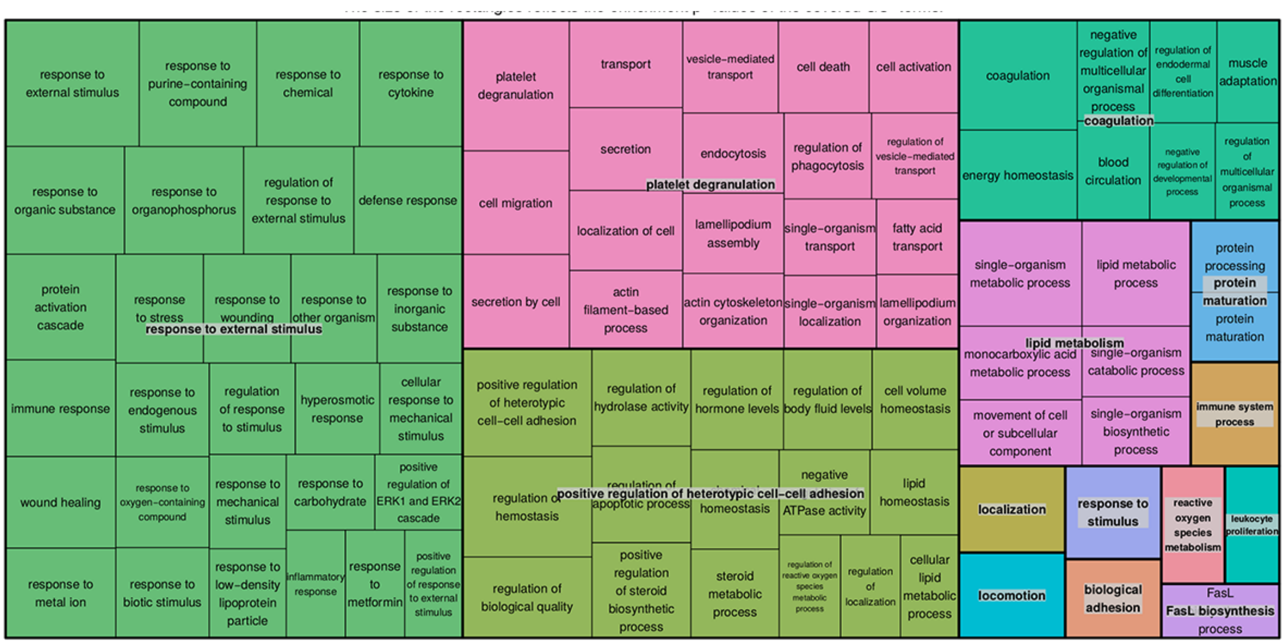

Figure S4. REVIGO treemap of gene ontology processes that are significantly enriched (FDR<0.05) for gene ontology processes in rat liver. Each rectangle is a single cluster representative. The representatives are joined into superclusters of loosely related terms, visualized with different colors. The size of the rectangles reflects the enrichment p-values of the covered GO-terms.

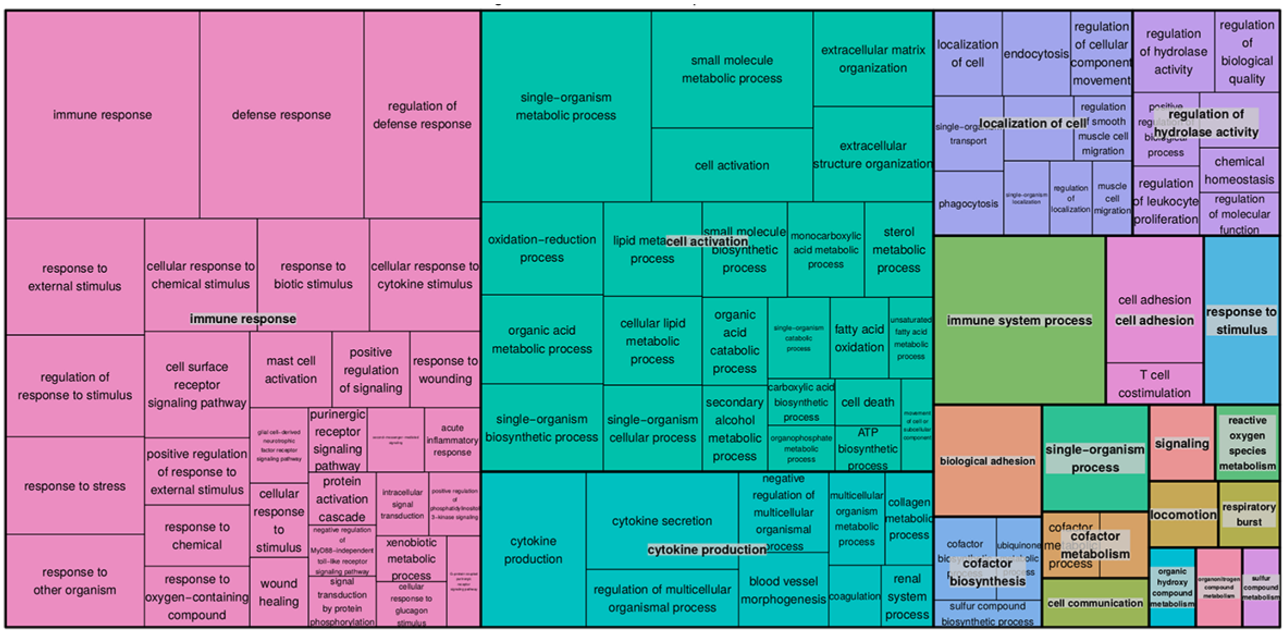

Figure S5. REVIGO treemap of gene ontology processes that are significantly enriched (FDR<0.05) for gene ontology processes in rat skin. Each rectangle is a single cluster representative. The representatives are joined into superclusters of loosely related terms, visualized with different colors. The size of the rectangles reflects the enrichment p-values of the covered GO-terms.

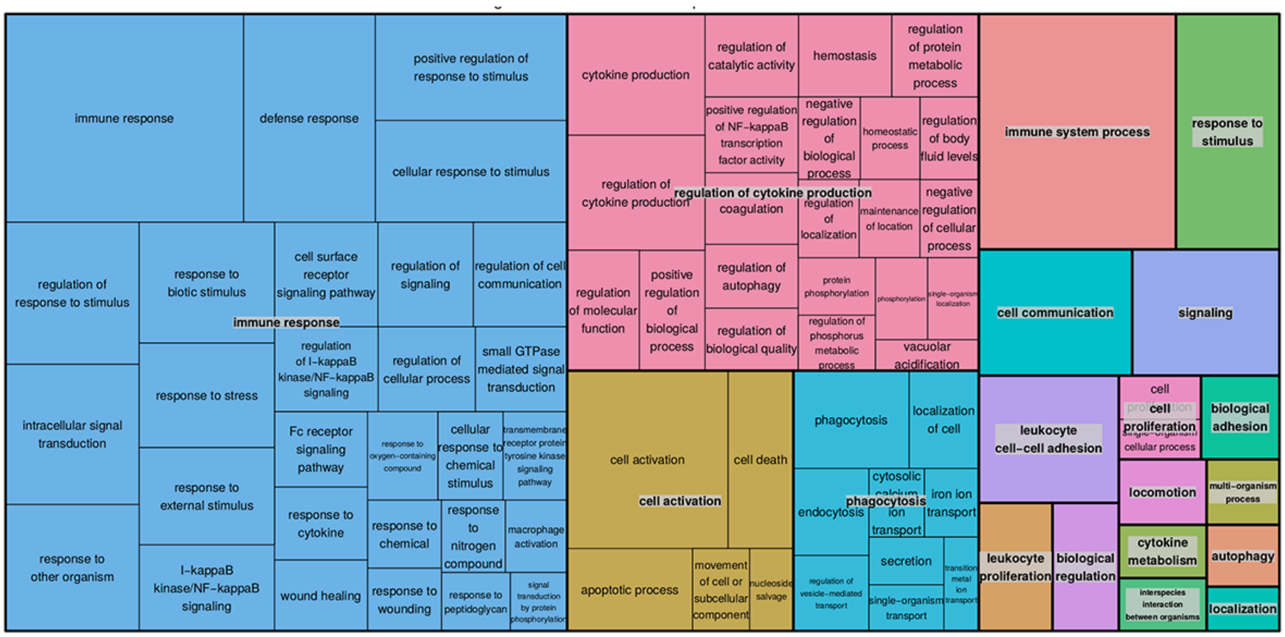

Figure S6 REVIGO treemap of gene ontology processes that are significantly enriched (FDR<0.05) for gene ontology processes in giant mole-rat blood. Each rectangle is a single cluster representative. The representatives are joined into superclusters of loosely related terms, visualized with different colors. The size of the rectangles reflects the enrichment p-values of the covered GO-terms.

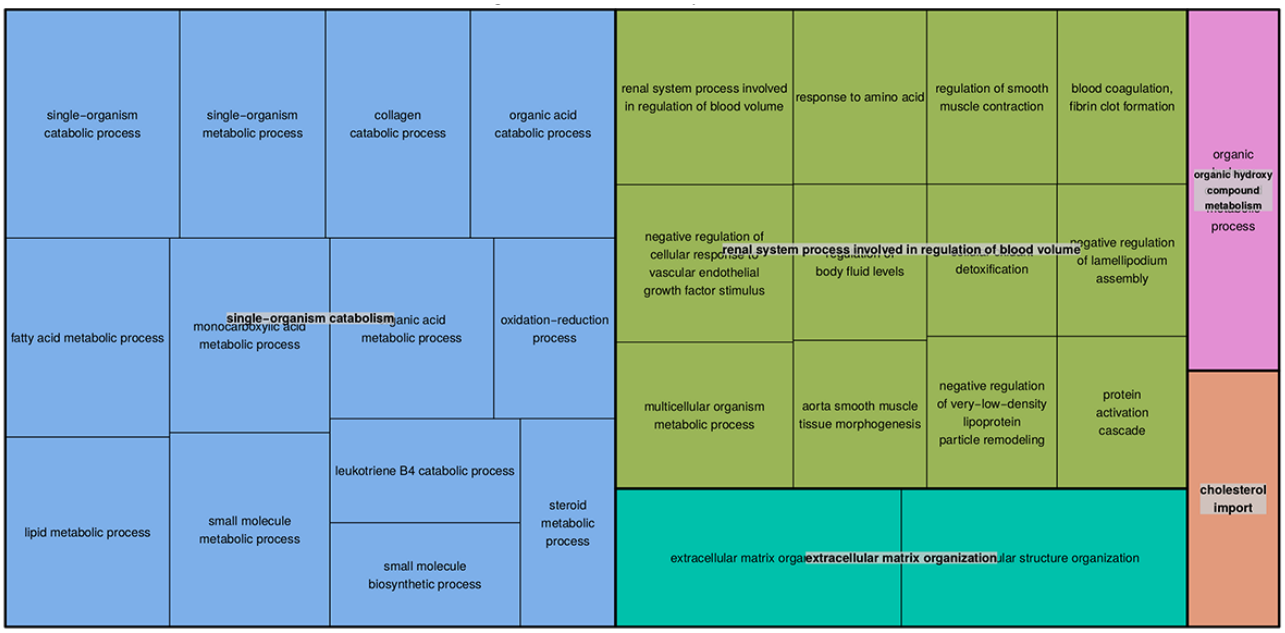

Figure S7. REVIGO treemap of gene ontology processes that are significantly enriched (FDR<0.05) for gene ontology processes in giant mole-rat heart. Each rectangle is a single cluster representative. The representatives are joined into superclusters of loosely related terms, visualized with different colors. The size of the rectangles reflects the enrichment p-values of the covered GO-terms.

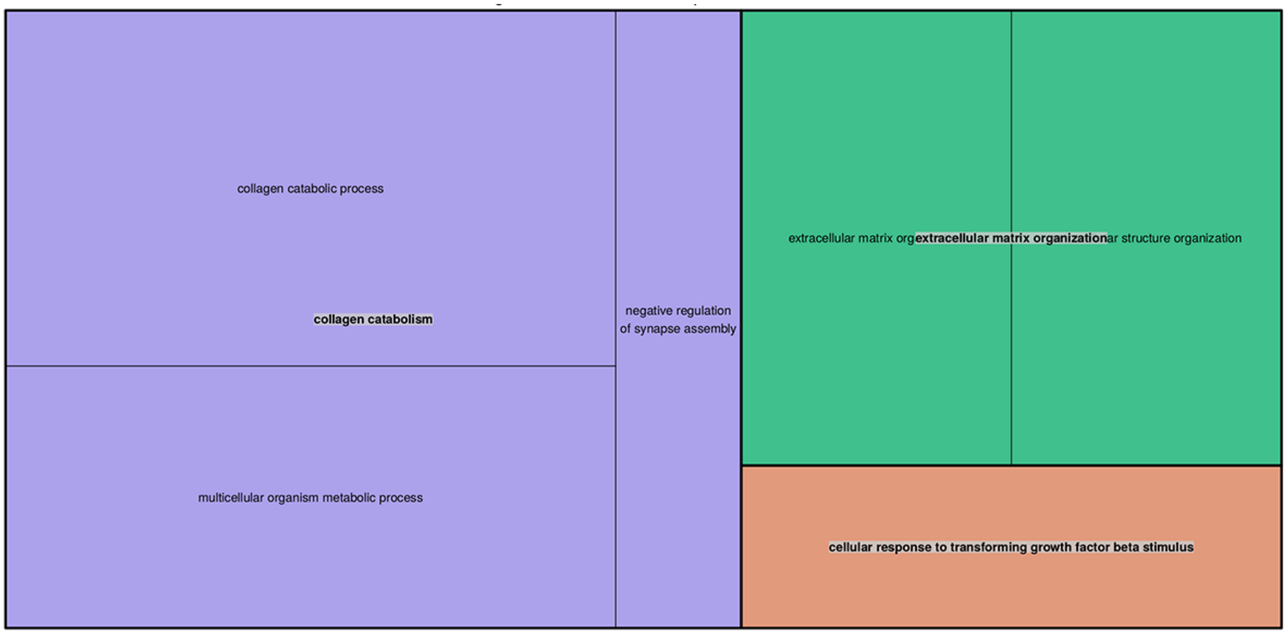

Figure S8. REVIGO treemap of gene ontology processes that are significantly enriched (FDR<0.05) for gene ontology processes in giant mole-rat kidney. Each rectangle is a single cluster representative. The representatives are joined into superclusters of loosely related terms, visualized with different colors. The size of the rectangles reflects the enrichment p-values of the covered GO-terms.

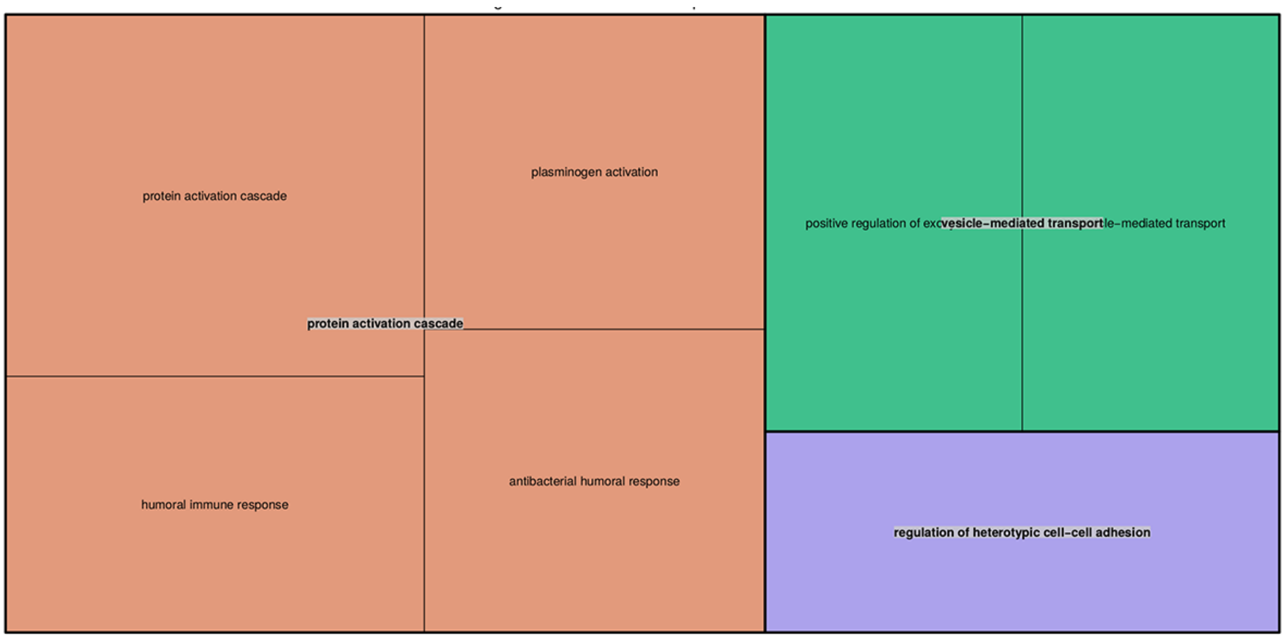

Figure S9. REVIGO treemap of gene ontology processes that are significantly enriched (FDR<0.05) for gene ontology processes in giant mole-rat liver. Each rectangle is a single cluster representative. The representatives are joined into superclusters of loosely related terms, visualized with different colors. The size of the rectangles reflects the enrichment p-values of the covered GO-terms.

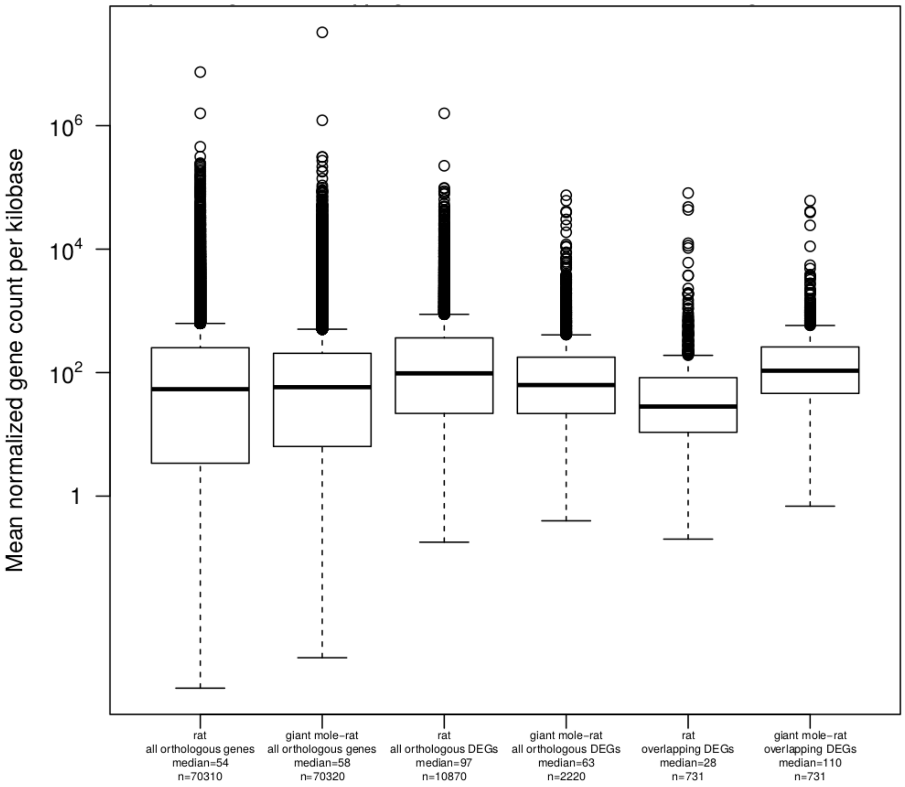

Figure S10. Expression levels of examined genes. Gene counts were first normalized for overall read number per sample. Then, separately for each tissue, across samples of, both, young and old animals, mean gene counts per kilobase transcript length were determined. Whiskers extend to the most extreme datum within 1.5 times inter quartile range. DEGs - differentially expressed genes. Overlapping DEGs - DEGs found, both, in rat and giant mole-rat.
